# Supplementary material for: Lactiplantibacillus plantarum GOLDGUT-HNU082 Alleviates CUMS-Induced Depressive-like Behaviors in Mice by Modulating the Gut Microbiota and Neurotransmitter Levels
Source: Foods. 2025 Feb 26;14(5):813. doi: 10.3390/foods14050813 (PMC11898433; doi:10.3390/foods14050813)
Supplement: Supplementary file 1 [file foods-14-00813-s001.zip › foods-3485506-supplementary.pdf]

Supplementary information

Table S1. Chronic unpredictable mild stress protocol.

Fig S1. Effects of Lp082 on neuron-related markers in the CA1 region of the hippocampus in CUMS model mice.

Fig S2. Effects of Lp082 on neuron-related markers in the CA3 region of the hippocampus in CUMS model mice.

Table S1. Chronic unpredictable mild stress protocol

	Week 1	Week 2	Week 3	Week 4
Monday	Confinement (9 h-15 h)	Stop the flash (9 h)	Return to normal light-dark cycle (9 h); cold water bath at 4°C for 5 min	Stop confinement (9 h); cold water bath at 4°C for 5 min
	Cage tilt 45° (15 h-21 h)	Confinement (14 h-21 h)	Damp sawdust (14 h-21 h)	Water deprivation and flash of light (19 h-)
Tuesday	Damp sawdust (9 h-17 h)	Cold water bath at 4°C for 5 min; water deprivation (9 h-21 h)	Fasting and water deprivation (9 h-21 h)	Stop the water ban and flashing; Cage tilt 45° (10 h-22 h)
	Illumination (19 h-)	Cage tilt 45° (21h-)	Cage tilt 45° (21 h-)	No sawdust and fasting (22 h-)
Wednesday	Return to normal light-dark cycle (9 h); cold water bath at 4°C for 5 min	Stop cage tilt 45° (9 h); Damp sawdust (9 h-19 h)	Stop cage tilt (9 h); confinement (10-20 h)	Confinement (8 h-20 h)
	Water deprivation (21 h)	Fasting (19 h-)	Confinement, flash of light (20 h-)	Cold water bath at 4°C for 5 min
Thursday	Stop the water ban (9 h); Cage tilt 45° (9 h-15 h)	Stop fasting (9 h); Confinement (9 h-17 h)	No sawdust, fasting and water deprivation (9 h-21 h)	Damp sawdust (9 h-20 h)
	Confinement (15 h-21 h)	No sawdust, illumination (19 h-)	Cold water bath at 4°C for 5 min	Illumination (20 h-)
Friday	Fasting (9 h-21 h)	Return to normal light-dark cycle (9 h); cold water bath at 4°C for 5 min	Damp sawdust (9 h-14 h)	Return to normal light-dark cycle (9 h); fasting for 24 h (9 h)
	Damp sawdust (21 h-)	Flash of light, damp sawdust (21 h-)	Illumination (19 h)	Cage tilt 45° (17 h-)
Saturday	Replacement of sawdust (9 h); water deprivation (9 h-21 h)	Stop the flash, replacement of sawdust (9 h); water deprivation (10 h-21 h)	Return to normal light-dark cycle (9 h); Cage tilt 45° (8 h-20 h)	Stop cage tilt (9 h); water deprivation for 24 h (9 h)
	Illumination (21 h-)	Cage tilt 45° (15 h-)	Fasting and flash of light (20h-)	Flash of light (19 h-)

Sunday	Return to normal light-dark cycle (9 h); Cage tilt 45° (10 h-20 h)	Stop cage tilt 45° (9 h); Confinement (9 h-19 h)	Stop fasting and flashing (9 h); damp sawdust (9 h-20 h)	Stop flashing (9 h); confinement (10 h-22 h)
	Fasting, flash of light (21 h-)	Illumination (20 h-)	Confinement (20 h-)	No sawdust and fasting (14 h-)

Repeated once for a total of 8 weeks

Expand the data legend

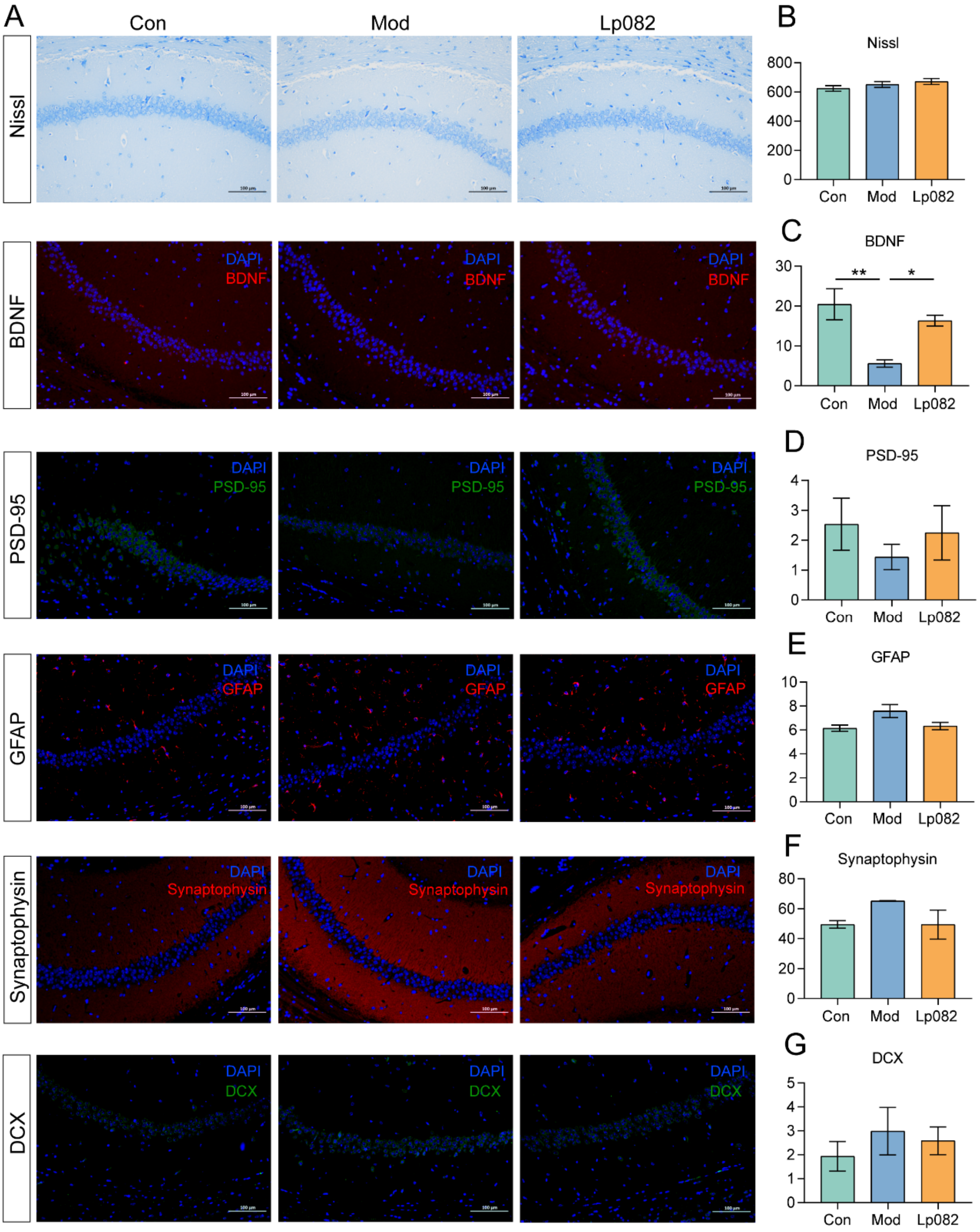

Figure S1. Effects of Lp082 on neuron-related markers in the CA1 region of the

hippocampus in CUMS model mice. (A) Nissl staining was used to observe the condition of neurons in the hippocampal CA1 region, and immunofluorescence staining showed the expression of BDNF, PSD-95, GFAP, Synaptophysin and DCX. (B-G) Percentage of positive expression region of the above markers in the CA1 area of hippocampus: Nissl staining (B), BDNF (C), PSD-95 (D), GFAP (E), Synaptophysin (F), and DCX (G). * Data are expressed as mean ± SEM. *$P < 0.05$, **$P < 0.01$, ***$P < 0.001$.

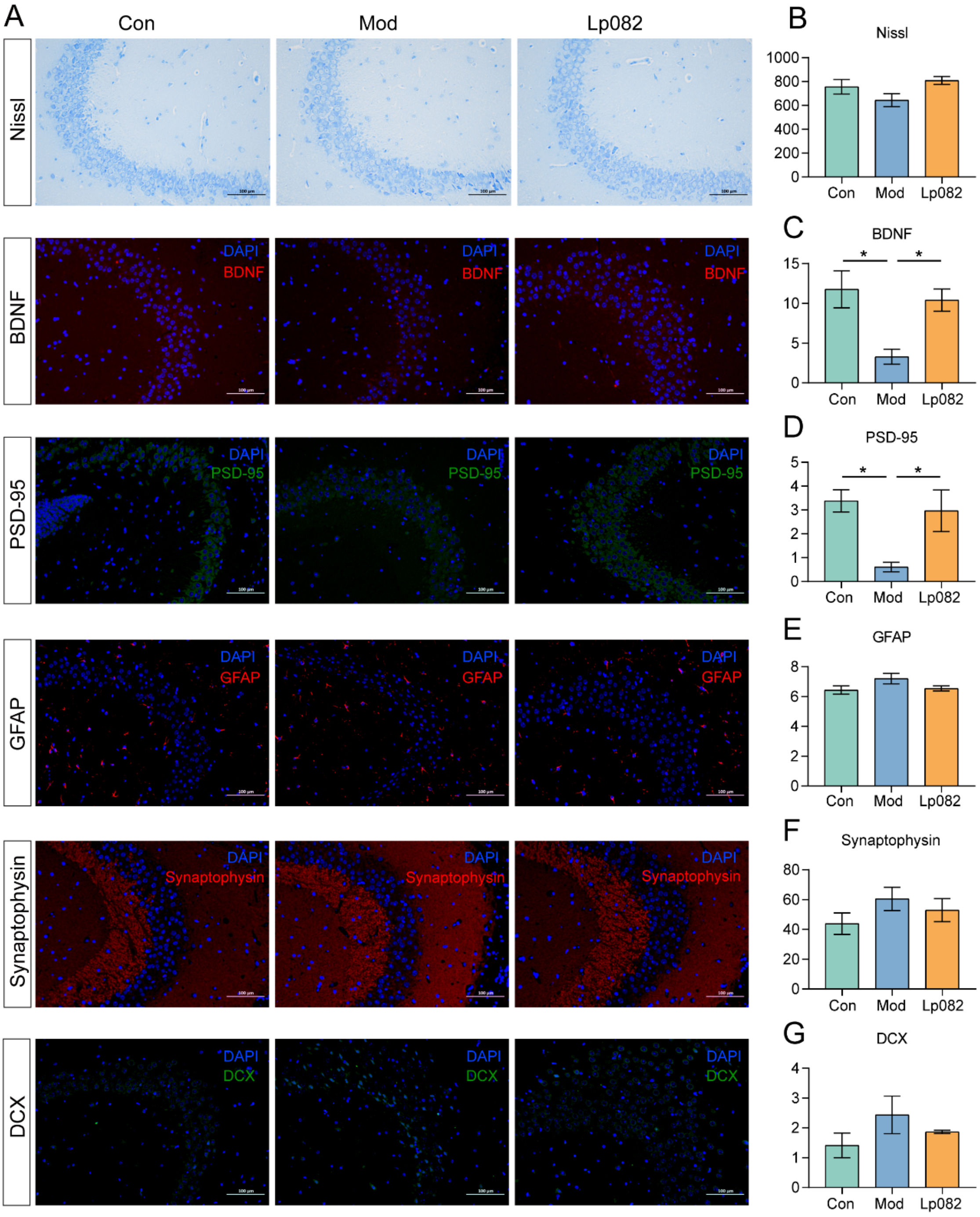

Figure S2. Effects of Lp082 on neuron-related markers in the CA3 region of the hippocampus in CUMS model mice. (A) Nissl staining was used to observe the condition of neurons in the hippocampal CA3 region, and immunofluorescence staining showed the expression of BDNF, PSD-95, GFAP, Synaptophysin and DCX. (B-G) Percentage of positive expression region of the above markers in the CA3 area of hippocampus: Nissl staining (B), BDNF (C), PSD-95 (D), GFAP (E), Synaptophysin (F), and DCX (G). * Data are expressed as mean ± SEM. *$P < 0.05$, **$P < 0.01$, ***$P < 0.001$.
